# Pediatric chronic hand eczema: Epidemiology, clinical presentation, and management

**DOI:** 10.1016/j.jdin.2023.02.008

**Published:** 2023-02-27

**Authors:** Michael A. Haft, Helen H. Park, Stephanie S. Lee, Jessica M. Sprague, Lawrence F. Eichenfield

**Affiliations:** a Division of Pediatric and Adolescent Dermatology, Rady Children’s Hospital, San Diego, California; b Department of Dermatology, University of California San Diego School of Medicine, San Diego, California; c Department of Pediatrics, University of California San Diego School of Medicine, San Diego, California

**Keywords:** adolescent, child, childhood, children, chronic, dermatitis, eczema, hand, literature, manus, pediatric, persistent, recalcitrant, review, skin, summary, teenage, teenager, questions, young

## Abstract

Chronic hand eczema (CHE) is persistent inflammatory dermatitis that may significantly affect the quality of life, with psychosocial effects, impact on school, work, and leisure activities, influence on socioeconomic status, and high health care costs. Pediatric-CHE (P-CHE) has a high prevalence yet has not been extensively studied in children and adolescents. There is minimal published data on P-CHE in North America, and no specific management guidelines. Limited prevalence data show broad ranges (0.9%-4.4%) in preschool and school children, with 1 study stating up to 10.0% 1-year prevalence for ages 16 to 19 years. Atopic dermatitis and allergic contact dermatitis appear important in the pathogenesis of this disease process, although there is limited pediatric data assessing disease associations and no standardized methodology for evaluating this disorder. Given the potential life-changing consequences of P-CHE, further research into this disease process is warranted to help generate best therapeutic practices and minimize this disease process’ morbidity in adulthood.

**Capsule Summary**

• Pediatric chronic hand eczema has a high prevalence yet has not been extensively studied in children and adolescents with no guidelines on its management.
• This review on pediatric chronic hand eczema highlights major findings in the literature and supports the need for further investigation of this life-changing disease process.

## Introduction

Chronic hand eczema (CHE) is defined by Diepgen et al^1^ as hand eczema with symptoms persisting for >3 months or with symptoms returning twice or more within 12 months. CHE significantly affects the quality of life, has financial and psychosocial consequences, which include job loss and high health care burdens, and can harm self-esteem and interpersonal relationships.^2^, ^3^, ^4^ The published literature on CHE in children and adolescents is limited. We evaluated and summarized pediatric chronic hand eczema (P-CHE) epidemiology, risk factors, disease associations, clinical presentations, severity classifications, diagnostic assessment, and therapeutic interventions. Knowledge gaps that might drive future research were identified.

## Materials and methods

A systematic search of PubMed, Embase, and Cochrane databases was performed from inception to December 5, 2022, for studies using search terms *hand eczema, hand dermatitis, hand,* and *eczema* or *dermatitis* in children, restricted to English-language articles. Records were screened according to title and abstracts (985 records), duplicates removed (112), and article eligibility was determined by including primary literature or review articles, observational or controlled studies, scope, including patients aged 0 to 20 years, and a diagnosis of hand dermatitis or eczema, yielding 31 manuscripts.

## Prevalence in children and adolescents

Multiple investigations have found that P-CHE is common, with a lifetime prevalence of 6.5% to 13.3% and a 1-year prevalence of 5.2% to 10.0% (Table I).^5^, ^6^, ^7^, ^8^, ^9^, ^10^ Figures of prevalence in children vary, with low prevalence at younger ages. Wang et al^10^ found that the median age of the first occurrence of hand eczema in children was 12 years.^10^ Grönhagen et al^6^ reported hand eczema incidence rates of approximately 0.9% per year in children aged 0 to 5 and 6 to 11 years and approximately 1.6% in children aged 12 to 16 years. Yngveson et al^9^ reported the point prevalence of hand eczema to be 3.9% (95% CI, 2.9%-5.0%) in grade 1 students and 4.4% (95% CI, 3.3%-5.5%) in grade 3 students in Sweden, although differences in data sets were not statistically significant. Much lower childhood lifetime prevalence data was reported by Crane et al^11^ (0.012%); however, the Depigen CHE diagnostic criteria were not used. Overall prevalence data correlate with general population data in both children and adults that demonstrate a lifetime prevalence of approximately 15% globally.^12^

**Table I:** Study data on the prevalence of hand eczema in pediatrics

Source	Setting	Study design (yrs)	Age of study participants included (yrs)	No. of total participants	No. of females	No. of males	No. of participants reporting			No. of females reporting		No. of males reporting	
							Lifetime prevalence of hand eczema (%)	1-yr prevalence of hand eczema (%)	Current hand eczema (%)	Lifetime prevalence of hand eczema (%)	1-yr prevalence of hand eczema (%)	Lifetime prevalence of hand eczema (%)	1-yr prevalence of hand eczema (%)
Grönhagen et al,^6^ 2014	Sweden; Birth registry	Birth cohort (1994-2012)	0-16	2927	1494	1433	284 (9.7)	152 (5.2)	NR	168 (11.2)	95 (6.4)	116 (8.1)	57 (4.0)
Johannisson et al,^7^ 2013	Sweden; 4 schools	Prospective Cohort (1995)	16-19	1516	857	659	202 (13.3)	NR	NR	139 (16.2)	NR	63 (9.6)	NR
Mortz et al,^8^ 2001	Denmark; 40 schools	Cross-sectional (1995-1997)	12-16	1438	713	725	133 (9.2)	105 (7.3)	46 (3.2)	87 (12.2)	72 (10.1)	46 (6.3)	33 (4.6)
Wang et al,^10^ 2021	Germany; 4 regions	Cross-sectional (2012-2014)	15	1468	715	753	153 (10.4)	NR	NR	91 (12.7)	NR	62 (8.2)	NR
Yngveson et al,^9^ 1998	Sweden; 4 schools	Cross-sectional (1995)	16-19	2572	1314	1258	NR	257 (10.0)	108 (4.2)	NR	322 (12.5)	NR	188 (7.3)

*No.*, Number; *NR*, not reported; *Yr.*, year.

Most studies report that female children are more affected than males, carrying a lifetime prevalence of 11.2% to 16.2% (vs 6.3%-9.6%) and a 1-year prevalence of 6.4% to 12.5% (vs 4.0%-7.3%) (Table I).^6^, ^7^, ^8^, ^9^, ^10^ Two studies have reported higher rates of adult women developing hand eczema before the age of 20 years (35% and 50%) than men (27% and 42%),^13^^,^^14^ and Röhrl and Stenberg^15^ observed a positive relationship between hand eczema and female sex (Table II).^8^^,^^10^^,^^16^^,^^17^ However, 2 recent robust investigations did not find statistically significant odds for P-CHE by sex (Table II).^10^^,^^16^ Conflicting sex-difference data in children contrast with sex-stratified broader population data demonstrating greater prevalence in the female population.^12^

**Table II:** Study data on risk factors for pediatric hand eczema

Source	Setting	Study design (yrs)	Age of study participants included (yrs)	No. of total participants	Odds ratio of association between				
					Female sex and HE (95% CI)	AD and HE (95% CI)	Asthma and HE (95% CI)	Allergic rhinitis and HE (95% CI)	Nickel allergy and HE (95% CI)
Grönhagen et al,^17^ 2015	Sweden; Birth registry	Birth cohort (1994-2012)	0-16	2927	NR	3.7 (2.0-7.0) (*P < .*01)	1.5 (0.8-2.5) (*P = .*2), 1.2 (0.6-2.1) (*P = .*6)†	NR	NR
Mortz et al,^8^ 2001	Denmark; 40 schools	Cross-sectional (1995-1997)	12-16	1438	NR	5.61 (3.81-8.25) (*P < .*001)	1.58 (1.01-2.46) (*P* < .05) (insignificant after Bonferroni correction)∗	NR	NR
Röhrl and Stenberg,^15^ 2010	Sweden; 11 schools	Cross-sectional (2000-2004)	14-24	7543	2.0 (1.3-3.2)	4.5 (3.3-6.1)	1.48 (1.04-2.09)	1.8 (1.3-2.5)	1.7 (1.1-2.7)
Silverberg et al,^16^ 2021	USA, Canada; >20 clinics	Retrospective (2000-2016)	0-18	1634	0.525 (0.497-0.554) (*P = .*6341)	0.989 (0.684-1.431) (*P = .*9550)	0.622 (0.378-1.023) (*P = .*0615)	0.782 (0.511-1.197) (*P = .*2578)	0.539 (0.349-0.832) (*P = .*00525)
Wang et al,^10^ 2021	Germany; 4 regions	Cross-sectional (2012-2014)	15	1468	1.5 (0.9-2.6) (*P = .*090)	1.8 (1.1-2.8) (*P = .*019)	NR†	1.4 (0.8-2.5) (*P = .*250)	NR

*AD*, Atopic dermatitis; *HE*, hand eczema; *No.*, number; *NR*, not reported; *Yrs*., years.

∗ Also included allergic rhinitis in their calculation.

† Not reported in multivariable logistic regression analysis.

Although these analyses use large subject populations, these investigations carry a number of issues, including recall bias, overestimating the number of participants with hand eczema and limiting most data sets to one city in one European nation.^5^, ^6^, ^7^, ^8^, ^9^, ^10^^,^^13^, ^14^, ^15^ Thus, they may not reflect P-CHE’s global epidemiology.

## Symptoms, distribution, and morphology

Symptoms and signs of CHE include itch, redness, scaling, oozing, crusting, and burning pain.^18^, ^19^, ^20^ Although symptoms may be important in assessing P-CHE severity and as potentially measurable parameters in clinical studies, there is minimal data on signs and symptoms in children and adolescents. Mortz et al^8^ found that children with hand eczema commonly report pruritus (82.7%), erythema (62.4%), and dry skin with scaling (54.1%). Simonsen et al^21^ found 26.2% of parents of children with hand eczema reported moderate-to-severe burning of the hands, 23.2% with moderate-to-severe pruritus, 12.6% with moderate-to-severe pain, and 6.5% with sleep disturbance. Five investigations reviewed the distribution of lesions in children with hand eczema and found areas of involvement to be variable. Depending on the study, the most commonly reported locations were dorsal aspect of the hands,^5^ finger webs or fingers,^8^^,^^22^ palms,^23^ fingertips, or diffuse hand involvement (Table III).^5^^,^^8^^,^^22^, ^23^, ^24^ Small data sets and reliance on self-reporting may explain the inconsistencies between studies.

**Table III:** Study data on the physical distribution of hand eczema in pediatrics

Source	Setting	Study design (yrs)	Age of study participants included (yrs)	No. of total participants	No. of participants with lifetime prevalence of hand eczema (%)	Out of all participants reporting lifetime prevalence of hand eczema, No. of those with:				
						Hand diffusely affected (%)	Fingers/finger-webs/lateral fingers (%)	Dorsal hands (%)	Palms (%)	Fingertips (%)
Dotterud & Falk,^5^ 1995	Norway; Multiple schools	Cross-sectional (1995)	7-12	551	36 (6.5)	NR	NR	14 (38.9)	1 (2.8)	NR
Lee et al,^22^ 2001	South Korea; 1 hospital	Cross-sectional (1997-1998)	0.5-12	108	62 (57.0)	NR	NR∗	38 (61.3)	48 (77.4)	NR
Mortz et al,^8^ 2001	Denmark; 40 schools	Cross-sectional (1995-1997)	12-16	1438	133 (9.2)	NR	86 (64.7)	68 (51.1)	22 (16.5)	NR
Ortiz-Salvador et al,^24^ 2018	Spain; 1 hospital	Retrospective observational (1996-2016)	0-16	389	42 (10.8)	12 (28.6)	4 (9.5)	5 (11.9)	9 (21.4)	12 (28.6)
Toledo et al,^23^ 2011	Spain; 11 hospitals	Retrospective multicenter (2005-2009)	0-15	480	111 (23.1)	19 (17.1)	15 (13.5)	7 (6.3)	29 (26.1)	14 (12.6)

*No.*, Number; *NR,* not reported; *Yrs*., years.

∗ Separately reported affected dorsal and ventral surfaces of fingers of right and left hands.

Few investigations assessed P-CHE severity. One study of 133 children who reported symptoms of hand eczema in the last 12 months found that 44% lacked any signs or symptoms at the time of evaluation, whereas 13% had moderate disease and 14% severe disease using the Hand Eczema Extent Score. Researchers did not specify how many investigators examined the hands of participants, resulting in possible measurement bias.^6^ Another study of 9 children with P-CHE found an average Hand Eczema Severity Index score correlating to severe disease before initiation of alitretinoin therapy; however, there was selection bias because all children failed multiple therapies before starting alitretinoin.^25^^,^^26^

These 6 studies constitute the bulk of the literature on the signs, symptoms, disease course, and outcomes in pediatrics. Hand eczema studies in adults show that the disease process presents with edema, erythema, or vesiculation in its acute form, and fissuring, scaling, or crusting chronically.^19^ In a twin cohort study based in Denmark, of those with hand eczema, 52.3% reported scaling, 51.4% reported erythema, 29.7% reported fissuring, and 20.7% reported vesicles. Of the 77 adults clinically examined, 47.7% had findings on the fingers (excluding fingertips), 35.1% on the palms of hands, 30.6% on the fingertips, and 24.3% on the dorsal aspect of the hands.^27^ Comparisons of these adult and pediatric data sets are insufficient given the small data sets and reliance on self-reporting in some investigations.

Unanswered questions remain regarding P-CHE’s presentation. Like the data available on prevalence, the published data are limited to Northern Europe. Courses and symptom complex are not well categorized in the pediatric population. Further research into the signs and symptoms of this disease presentation in children and adolescents would be useful.

## Risk factors and diagnoses

Multiple studies have suggested that CHE is strongly associated with atopic dermatitis (AD) in children. In one study, 43.7% of children aged 0 to 2 years and 54.1% aged 3 to 12 years with AD had hand eczema, although chronicity or duration were not noted.^28^ Two investigations from Mortz et al^8^^,^^29^ and one from Grönhagen et al^17^ found odds ratios of 3.7–5.61 between childhood hand eczema and AD (Table II). In an evaluation of pediatric patients referred for patch testing to the North American Contact Dermatitis Group, children with hand eczema were more likely to have a diagnosis of AD than adults.^16^ Mortz et al^30^ in 2015 found an odds ratio of 4.3 between hand eczema in childhood and persistent AD in adulthood, whereas Wang et al^10^ in 2021 calculated an adjusted odds ratio of 1.8 between previous diagnosis of AD and lifetime incidence of hand eczema in children aged 15 years. Data regarding AD age of onset and hand eczema risk are conflicting. Wang et al^10^ reported a statistically significant adjusted odds ratio of 1.8 between the early age of onset of AD, independent of the diagnosis of AD itself, and pediatric hand eczema. However, Grönhagen et al^17^ found no differences between the odds ratios of hand eczema and AD at different onset ages of AD. With regards to hand eczema’s relationship to generalized eczema, Silverberg et al^16^ found that hand eczema was associated with lower proportions of generalized dermatitis. Although filaggrin mutations are believed to be among the strongest risk factors for developing AD,^31^ a logistic regression analysis performed by Lagrelius et al^32^ found no statistically significant odds ratio between filaggrin gene mutations and P-CHE.

The data on the relationship of inhalant allergy to pediatric hand eczema are inconsistent. Röhrl and Stenberg^15^ found significant associations between hand eczema and asthma as well as hand eczema and allergic rhino conjunctivitis (Table II); however, memory bias and use of invalidated questions in this investigation may have skewed the results. Mortz et al, Wang et al, Silverberg et al, and Grönhagen et al found no such links (Table II).^8^^,^^10^^,^^16^^,^^17^ Given this discrepant data, these relationships must be further investigated.

P-CHE may also be associated with allergic contact dermatitis (ACD).^29^^,^^33^ One investigation of children with AD found that 43.8% of children with hand and/or foot eczema had contact allergy vs 16.0% of children without hand or foot dermatitis.^33^ Another study found that 35.7% of patients with ACD had hand involvement.^34^ Patch testing of children with hand eczema revealed that the most common or most relevant allergens associated with the disorder includes nickel, methylchloroisothiazolinone, and methylisothiazolinone (MI) (which are commonly found in cosmetic, hygiene, and household products), and cobalt.^16^^,^^22^^,^^35^, ^36^, ^37^, ^38^, ^39^, ^40^ Nickel and MI sensitization stand out as major risk factors for P-CHE, with other allergens less common. Adult population data carries similar findings, as one report found the most frequent sensitizers in adults with hand eczema to be nickel, methylchloroisothiazolinone and MI, cobalt chloride, and fragrance mix I.^41^

The evidence of irritant contact dermatitis’ influence on P-CHE is less clear. In 2020 and 2021, Simonsen et al^21^^,^^42^ found that 26.2% of children aged 0 to 7 years and 36.3% of children aged 5 to 13 years who were investigated developed hand eczema after strict hand hygiene protocols on return to daycare or school in the middle of the COVID-19 pandemic, with frequency of handwashing, female gender, and history of AD associated with an increased risk of developing hand eczema. However, in a 2017 study by Meding et al,^43^ investigators found no association between pediatric hand eczema and hand-water exposure.

P-CHE has several coupled diagnoses. Two studies found the most common final diagnoses of children with P-CHE to be ACD, AD, and vesicular (dyshidrotic) eczema (Table IV).^22^^,^^23^ Another found that the most common diagnoses for children with CHE referred for patch testing to as ACD, AD, and irritant contact dermatitis (ICD) (Table IV).^16^ This suggests that ACD and AD are commonly associated with CHE in childhood. The adult CHE literature presents some overlap in findings, with one analysis presenting the most common associated diagnoses as combinations of ICD, ACD, and vesicular eczema,^1^ suggesting that AD plays a greater role in P-CHE pathogenesis with ICD playing a greater role in adult CHE. In clinical practice, it appears that some children and adolescents have significant chronic hand dermatitis as part of a constellation of findings in active AD, whereas others have localized CHE, or predominate issues with CHE disproportionate to other issues with AD. We believe that the term CHE remains useful, with subcategories of etiology, including AD and ACD.

**Table IV:** Study data of diagnoses associated with pediatric hand eczema

Source	Setting	Study design (yrs)	Age of study participants included (yrs)	No. of total participants	No. of participants with lifetime prevalence of hand eczema (%)	No. of participants with hand eczema diagnosed with:				
						Atopic dermatitis (%)	Allergic contact dermatitis (%)	Irritant contact dermatitis (%)	Hyperkeratotic eczema (%)	Vesicular eczema (%)
Ortiz-Salvador et al,^24^ 2018	Spain; 1 hospital	Retrospective observational (1996-2016)	0-16	389	42 (10.8)	15 (35.7)	14 (33.3)	2 (4.8)	5 (11.9)	6 (14.3)
Silverberg et al,^16^ 2021	USA, Canada; >20 clinics	Retrospective (2000-2016)	0-18	1634	237 (14.5)	88 (37.1)	117 (49.4)	40 (16.9)	NR	10 (4.2)
Toledo et al,^23^ 2011	Spain; 11 hospitals	Retrospective multicenter (2005-2009)	0-15	480	111 (23.1)	32 (28.8)	40 (36)	17 (15.3)	NR	18 (16.2)

*No.*, Number; *NR,* not reported; *Yrs*., years.

Investigations show conflicting data regarding the influence of inhalant allergy and ICD on P-CHE. AD and ACD’s overlap with and impact on P-CHE are much clearer,^16^ and evidence demonstrates nickel and MI allergy’s influence on hand eczema in children. Further investigations need to elucidate the relationship between these and other risk factors for the development and persistence of CHE in childhood.

Many methods of diagnosis/classification of CHE^44^, ^45^, ^46^, ^47^ attempt to incorporate various combinations of morphology, etiology, and chronological progression, while major studies have found an insignificant association between classification and etiology.^45^^,^^46^^,^^48^

## Diagnostic testing, severity assessment, and therapeutics

P-CHE workup frequently includes patch testing with studies finding that anywhere from 14.5% to 28.0% of children referred for patch testing have hand eczema.^16^^,^^22^^,^^35^^,^^49^ In 2 studies, patch testing was reported to have clinical relevance of 78% in P-CHE and 76.2% in pediatric hand eczema,^22^^,^^50^ which was much higher than that reported in adult studies.^51^^,^^52^ The literature has not supported immunoglobulin E testing because no association has been found between positive specific immunoglobulin E during childhood and P-CHE.^17^

The use of standardized severity assessments is rare in the P-CHE literature, with one P-CHE study using the Hand Eczema Severity Index and Investigator Global Assessment^24^ and one study using the Hand Eczema Extent Score.^6^ Other evaluation measures, including the Dermatology Life Quality Index,^17^ Quality of Life in Hand Eczema Questionnaire,^53^ and modified total lesion symptom score^54^ appear to only be executed in adult populations or in studies containing mixed populations of both children and adults.^55^

Studies evaluating topical or systemic medications for P-CHE are limited. In a retrospective analysis of 13 children who received systemic alitretinoin therapy, 9 were children with CHE. In this subgroup, 7 of 9 had moderate to excellent results on alitretinoin.^24^ In a retrospective review of 75 children receiving phototherapy for cutaneous conditions, 4 had severe hand eczema, of which 3 had clinical improvement after psoralen and UV-A therapy.^56^ In a 2019 systematic review of publications on hand eczema therapeutics performed by Christoffers et al,^57^ researchers could not find a single study on therapeutics exclusively in pediatrics. Most of the studies that excluded children and pediatric patients were not given their own subgroup analysis apart from adults in any article.

Although the literature lacks published data on P-CHE treatment, investigators from this article and the Pediatric Dermatology Research Alliance, performed a survey of pediatric dermatologist CHE experts. Surveyed respondents all use topical corticosteroids as first-line topical therapy with most choosing topical corticosteroids, topical calcineurin inhibitors, and topical phosphodiesterase-4 inhibitors as second-line agents. Systemic treatment use is rare, with most respondents reporting ≤5 patients treated for the indication of P-CHE. The most preferred systemic agent for P-CHE was dupilumab, followed by methotrexate.^58^

No specific guidelines exist for P-CHE management, although there are published guidelines and consensus statements for the management of CHE based on adult data.^59^^,^^60^ The European Society of Contact Dermatitis produced updated management guidelines for hand eczema in 2022, recommending the use of patch testing in all patients with CHE. Other recommendations included skin prick testing, microbial testing, and cutaneous biopsy when deemed appropriate. However, there is significant disagreement among experts concerning the utility of patch testing irrespective of morphology and location, predictive value of testing, and cost effectiveness.^48^ Management includes prevention and use of therapeutics from emollients and topical steroids to systemic agents, such as oral alitretinoin (approved for CHE in Europe and the United Kingdom) or cyclosporin.^61^ Recent literature highlights the use of emerging and investigational systemic agents, including biologic agents and JAK inhibitors for CHE in adults.^62^

The lack of scoring systems, published data on therapeutics, and management guidelines focused on the pediatric population is troubling given the life-altering potential of this disorder.^4^ Utilization of standardized metrics of disease severity, quality of life, and treatment response could assist in determining the comparative efficacy of various interventions and guide the development of best practice guidelines.

## Future directions and significance of further investigations

There remain wide knowledge gaps in epidemiology, presentation, risk stratification, diagnosis, and management of CHE in pediatric populations. Most published studies are limited to patients in Northern Europe. Most of the data on CHE epidemiology are based on adult patients.^12^ Although many adults report hand eczema onset in childhood, few studies investigated the characteristics of this disease in the pediatric population, including assessing the percentage of children with hand eczema who have AD. Even fewer studies have explored scoring systems in the assessment or therapeutic management of P-CHE.

Numerous questions remain in all domains of this disorder in children and adolescents: What is the course of hand eczema in childhood and adolescence compared with adulthood? How does hand eczema in pediatrics progress from acute to chronic disease? What percentage of those affected have active AD or other inflammatory skin conditions? What are other risk factors for disease development? What classification systems are ideal? Why is there little consensus on features and testing? When would providers use certain tests for disease workup and treatments for disease management? How do clinicians assess the treatment response? How does therapeutic data correlate with the severity or quality of life scoring?

Early recognition and treatment of this disease process in childhood may minimize the disease impact, decrease health care burden, and improve the quality of life.^4^ Further investigations into the epidemiology of P-CHE onset and course, disease associations, comorbidities, and therapeutics are important to determining best practices to allow for comprehensive and successful management. With the ongoing development of new topical and systemic agents for CHE as well as for AD, focused research on P-CHE is warranted.

## Conflicts of interest

Dr Eichenfield has served as an advisory board member, and/or speaker, consultant, or clinical trial investigator for AbbVie, Arcutis, Almirall, Arena, Aslan, Bausch, Castle, Dermavant, Eli Lilly, Forte, Galderma, Gelnmark/Ichnos, Incyte, Leo Pharma, Novartis, Pfizer, Regeneron, Sanofi Genzyme, UCB, and Valeant/Ortho Derm. Drs Haft, Park, Lee, and Sprague have no conflicts of interest to declare.
